# Gene profiling of B lymphoblastoid cell lines from DRESS patients after drugs incubation

**DOI:** 10.1186/2045-7022-4-S3-P123

**Published:** 2014-07-18

**Authors:** Sébastien Calbo, Xavier Camous, Baptiste Janela, Kim Périchon, Jinmiao Chen, Edmond Chua, Josephine Lum, Francesca Zolezzi, Michael Poidinger, Philippe Musette

**Affiliations:** 1 Inserm U905, Normandie Univ, France; 2 Singapore Immunology Network, A STAR, Singapore

## Background

Drug reaction with eosinophilia and systemic symptom (DRESS) is a severe drug-induced cutaneous reaction with visceral involvement and blood abnormalities associated with reactivations of herpes-virus family i.e., EBV, HHV-6, HHV-7 and CMV. Recently, we demonstrated that the immune response in DRESS, previously thought to be directed only against drug components, is also mediated by herpes-virus specific cytotoxic CD8+ T lymphocytes which home to the skin and visceral organs.

## Method

An in vitro model based on EBV-transformed B lymphoblastoid cells lines from DRESS patients and healthy donors were used to study the specific effect of drugs on gene expression. Gene expression profiles were obtained for cell lines treated or not by allopurinol, amoxicilline, sulfamethoxazole, valproic acid or carbamazepine.

## Results

Significant differential gene expressions were found in DRESS patients' cell lines in comparison with healthy donors' cell lines after sulfamethoxazole or valproic acid treatment. Ingenuity pathways analysis revealed that differentially gene expressed following sulfamethoxazole treatment were playing a role in antigen presentation, immune cell trafficking, proliferation and inflammatory response. We confirmed modified gene level expression at the protein level by flow cytometry. Finally, we performed a functional assay to address the role of sulfamethoxazole on immune response by setting up an in vitro proliferation assay.

## Conclusion

Our results suggest a new role for drugs in the pathogenesis of DRESS that could enhanced the immune response in vivo.

